# Symbiotic organisms search algorithm for the unrelated parallel machines scheduling with sequence-dependent setup times

**DOI:** 10.1371/journal.pone.0200030

**Published:** 2018-07-05

**Authors:** Absalom E. Ezugwu, Olawale J. Adeleke, Serestina Viriri

**Affiliations:** School of Mathematics, Statistics and Computer Science, University of Kwazulu-Natal, Westville Campus, Durban, South Africa; Universitas Kristen Petra, INDONESIA

## Abstract

This paper addresses the problem of makespan minimization on unrelated parallel machines with sequence dependent setup times. The symbiotic organisms search (SOS) algorithm is a new and popular global optimization technique that has received wide acceptance in recent years from researchers in continuous and discrete optimization domains. An improved SOS algorithm is developed to solve the parallel machine scheduling problem. Since the standard SOS algorithm was originally developed to solve continuous optimization problems, a new solution representation and decoding procedure is designed to make the SOS algorithm suitable for the unrelated parallel machine scheduling problem (UPMSP). Similarly, to enhance the solution quality of the SOS algorithm, an iterated local search strategy based on combining variable numbers of insertion and swap moves is incorporated into the SOS algorithm. More so, to further improve the SOS optimization speed and performance, the longest processing time first (LPT) rule is used to design a machine assignment heuristic that assigns processing machines to jobs based on the machine dynamic load-balancing mechanism. Subsequently, the machine assignment scheme is incorporated into SOS algorithms and used to solve the UPMSP. The performances of the proposed methods are evaluated by comparing their solutions with other existing techniques from the literature. A number of statistical tests were also conducted to determine the variations in performance for each of the techniques. The experimental results showed that the SOS with LPT (SOS-LPT) heuristic has the best performance compared to other tested method, which is closely followed by SOS algorithm, indicating that the two proposed algorithms’ solution approaches are reasonable and effective for solving large-scale UPMSPs.

## Introduction

The parallel machine scheduling problem (PMSP) is one of the most intensively studied problems in combinatorial optimization, probably because of its considerable theoretical interests and as a representative of many real world problems such as, production lines; hospital management systems (e.g. nurses or doctors’ scheduling problems); university management systems (e.g. timetabling scheduling problems); and shipping docks’, flow shops’ and job shops’ systems. The parallel machine environments consist of either similar or unrelated types of multiple numbers of machines on which jobs can be scheduled simultaneously. Because PMSP involves both machines assignment and jobs sequencing decisions [1], the problem is difficult to solve and is said to be NP-hard [2]. To successfully solve the PMSP, it is required to concurrently determine both assignment and sequencing policies for the available parallel machines and jobs [1]. The current proposal therefore, considers the integration of both policies into the solution search space of the newly proposed population based SOS optimization algorithm, which was initially introduced to solve continuous engineering optimization problems [3].

The PMSP, like its scheduling counterparts, has received wide attention in the literature with different solution approaches to the problem being proposed. Few exact solution methods have been proposed and applied to solve the PMSPs. Examples of these methods include the branch-and-bound algorithm [4–8] and the cutting plane algorithm [8]. One major limitation of the exact solution is that it is designed to solve some specific problems, which therefore limits its application area. However, several alternative metaheuristic algorithms have been proposed too, of which many have been successfully applied to solve the UPMSP. Some of the related population based metaheuristic algorithms include particle swarm optimization (PSO) algorithms (see [9–13]), ant colony optimization (ACO) algorithm (see [14–17]), and cuckoo search (CS) algorithm (see [18, 19]), artificial bee colony (BAC) algorithm (see [20], and genetic algorithm (GA) [21]. More so, relevant literatures on UPMSPs can be found in [22–26].

There are some existing studies that considered the use of metaheuristics to handle UPMSP with sequence dependent setup times. For example, in [21], a GA that includes a fast local search and a local search enhanced crossover operator was presented for solving the UPMSP in which machine and job sequence dependent setup times are considered. From the exhaustive computational and statistical analysis conducted by the authors, it can be concluded that the proposed method obtained an excellent performance compared to the rest of the evaluated methods in a comprehensive benchmark set of instances. In [16], an Ant Colony Optimization (ACO) algorithm for the UPMSP was proposed, and the preliminary results obtained showed better performance when compared with other existing techniques such as the partitioning heuristic (PH) presented in [4] and the tabu search (TS) algorithm presented in [15]. An extension of the ACO algorithm referred to as ACOII for UPMSP was similarly proposed by the same author in [14] with consideration to optimized parameters. The experimental results obtained showed that there were significant improvements in the solution qualities of the ACOII as compared to the results of the PH, TS, metaheuristic for randomized priority search (Meta-RaPS) and ACO algorithms. Similarly, in [27], a variant of the genetic algorithm called GADP that integrated a set of dominance properties to improve the solution quality of the UPMSP was proposed. The result obtained showed that by applying these dominance properties for a given sequence, a near-optimal solution can be derived. To further support this claim, the experimental results showed that the GADP was able to obtain all optimal solutions for small test problems, and outperformed the solutions yielded by the PH algorithm and other competing algorithms in both effectiveness and efficiency for larger test problems. In another research contribution, a hybrid artificial bee colony (HABC) algorithm was presented in [20] to solve the UPMSP with the objective of minimizing the makespan. The performance of HABC algorithm was evaluated by comparing its solutions to other state-of-the-art metaheuristic algorithms and the results obtained showed that the HABC outperformed these algorithms.

In [3], a new nature inspired and population based global optimization metaheuristic algorithm, known as the symbiotic organisms search, was reported. The algorithm is modeled based on the mode of relationship interactions among the various organisms that cohabit in the ecosystem. One key advantage and characteristic of the SOS is that it is parameter free and therefore, does not require any form of parameter fine-tuning. The only basic requirement of the algorithm is the initialization and setting of the algorithm’s number of function evaluation or generation. However, for the sake of this study, an additional parameter setting is required to make SOS suitable and adaptive for the current problem. The performance superiority of the SOS over other related population based algorithms such as the ACO, PSO, differential evolution and genetic algorithm on the optimization of twenty-six mathematical benchmark functions, was also reported in the same foundational paper presented in [3]. Since its introduction in the literature, SOS has attracted the attention of numerous researchers from different domains. This led to its application in different fields of studies, namely engineering [28, 29], power systems [30–32], cloud computing [33], and distribution scheduling [34, 35].

Inspired by the recent trend in the performance achievements and applications of the SOS algorithm into different research domains, in this work an improved SOS algorithm is developed to solve the unrelated parallel machine scheduling problem with the main objective of minimizing makespan. The method applied proceeded in two steps. First, the LPT rule, known to be the most appropriate dispatching rule for the problem at hand [36, 37], is used as a machine dispatching or an assignment heuristic to find partial optimal schedules for the problem of scheduling non-preemptive jobs on unrelated parallel machines with setup times. Second, all the partial schedules generated by the LPT are sequenced by using the SOS optimization algorithm to minimize the total setup costs. In addition, the proposed SOS algorithm is enhanced with an iterated local search mechanism that uses a random kick move. A random kick move is defined here as a series of random insertion moves or a series of random swap moves used for the implementation of the SOS solution generation procedures. To evaluate the performance of the SOS algorithm, an improved population based simulated annealing (PSA) algorithm is implemented and used to compare the efficiency of the proposed SOS algorithm in terms of the algorithm convergence speed and solution quality. Subsequently, to rigorously evaluate and present fair comparisons of the two proposed methods with other related approaches from the literature, the balanced UPMSP standard benchmark data available at [38] are used.

The technical contribution of this paper can be summarized into four parts. First, the applicability of the SOS algorithm to solve the UPMSP by incorporating an iterated local search strategy into it is demonstrated. Second, it is shown that the optimization speed of the proposed SOS scheduling method can be improved with better results when combined with a machine assignment heuristic, the LPT, as is the case in this paper. Third, it is demonstrated that the SOS algorithm is a good alternative solution technique for solving large scale PMSPs when compared to other well-known existing heuristics. Fourth, another main contribution is the implementation of a new solution representation and decoding procedure that is designed to make the SOS algorithm more suitable for solving the UPMSP. Finally, to the best of the researchers’ knowledge, this work is the first to apply an SOS metaheuristic algorithm to solve the UPMSP with Sequence-dependent setup times, which specifically served as the primary motivation for undertaking this study.

The rest of the manuscript is systematically organized as follows: The next section presents a brief description of the UPMSP. The details of the LPT machine assignment heuristic and the SOS algorithm are outlined afterwards. This is followed by the presentation of the proposed SOS optimization framework for the problem at hand, while the computational results and algorithms’ evaluation are presented subsequently. The last section concludes the paper and gives future research directions.

## Problem description

The scheduling problem of minimizing the total completion time on unrelated parallel machines examined in this paper is stated as a triplet: *P*_*m*_|*S*_*i*,*j*,*k*_|*C*_*max*_. The first field, *P*_*m*_, describes the unrelated parallel machine environment. Reference to unrelated machines means that the job processing time *P*_*i*,*k*_, which is the processing time of job *i* on machine *k*, depends on the machine to which they are assigned, and there is no relationship between machine speeds. The second field, *S*_*i*,*j*,*k*_, describes the scheduling constraints, which in this case are the sequence-dependent setup times, where the setup time required for job *i* when it succeeds job *j* on machine *k*, may be different from that required to set up job *i* if it precedes job *j* on the same machine such that *S*_*i*,*j*,*k*_ ≠ *S*_*j*,*i*,*k*_, for each *i*,*j* ∈ {0,1,…,*n*}. The third field, *C*_*max*_, is the objective function, which is the makespan (maximum completion time of a schedule *X* of *n* jobs).

**Fig 1:**
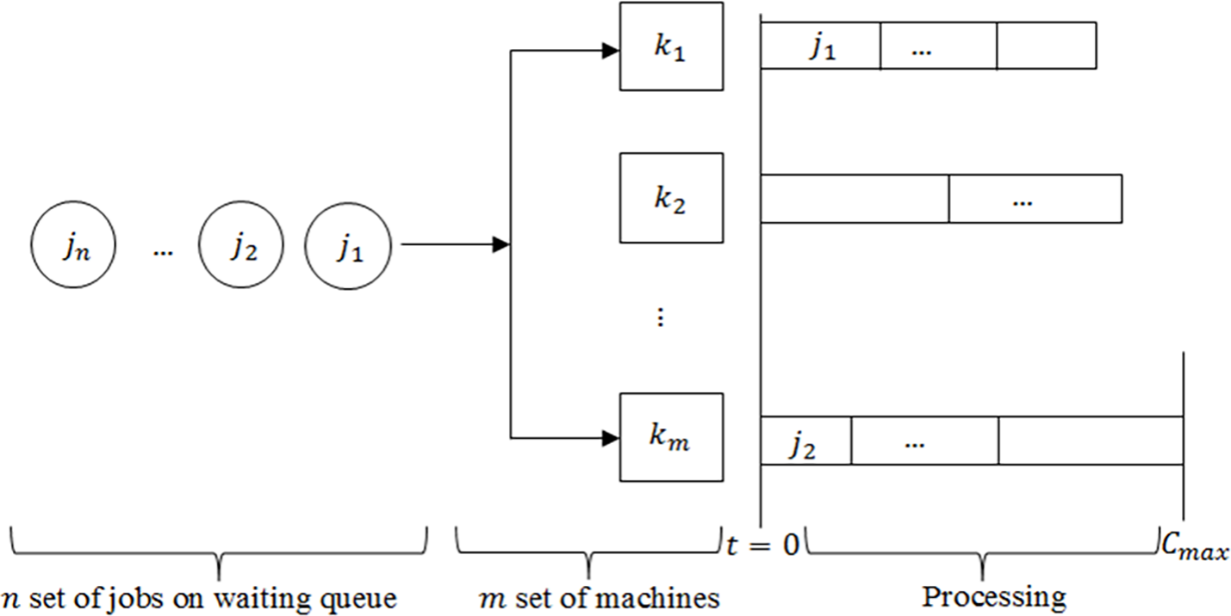
Parallel machine scheduling problem model.

Therefore, the scheduling model can be described as follows: Given a set of *n* independent jobs *I* = {1,2,…,*n*} with positive processing times *P*_*i*_, to be processed on a set of unrelated parallel machines *M* = {1,2,…,*m*}. All jobs are available for processing at time zero and once a job starts processing on any of the machines, it must reach completion. In other words, job preemption is not allowed. The objective of this paper therefore is to find a non-preemptive schedule *X* = {*X*_1_,*X*_2_,…,*X*_*n*_} on a set of unrelated parallel machines *k* (*k* = 1,2,..,*m*) of job *j* (*j* = 1,2,..,*n*), in such a way that the makespan is minimized. The parallel machine scheduling environment is illustrated in Fig 1.

## Scheduling heuristics

This section provides an introductory explanation on the LPT heuristic and SOS algorithm, followed by a more detailed description of each of these techniques with their applications to solve the PMSP. The LPT is a common dispatching heuristic employed to generate schedules for the PMSP, whereas the SOS is a new population based metaheuristic algorithm that has a wide range of applications in engineering and scientific computing. The scheduling method proceeds in two stages of assignment and sequencing, comprising the following:

- The LPT heuristic generates a set of initial partial assignment schedules, which must satisfy machine constraints with respect to load balancing, to minimize the total completion time.
- The SOS algorithm finds the sequence of the partial schedules to minimize the total setup time.

These techniques and their applications to solve the parallel machine scheduling problem entail the following:

### Longest processing time first heuristic

The LPT heuristic is a well-known dispatching heuristic that is often employed to determine a near-optimal schedule for the parallel machine scheduling problems [39, 40]. A dispatching heuristic assigns a priority index to every job in a waiting queue. However, other dispatching heuristics exist as well, including the earliest due date first (EDD) rule, shortest processing time first (SPT) rule, least slack time first (LST) rule, and longest remaining processing time first (LRPT) rule [39]. For a comprehensive review in this regard, reference is made to [40, 41]. The LPT heuristic was chosen for the scheduling model implementation, basically because of it guarantees high performance and simple implementation. According to this rule, jobs in the waiting queues are arranged in decreasing order of processing times. The jobs are then sorted in such a way that the ones with the largest values of processing times are given high priority to be scheduled on the parallel machines. Generally, the LPT commences with the creation of an empty schedule and iteratively generating a queue of non-scheduled jobs beginning with the job having the highest processing time, proceeding to the one with the least processing time. In this order, the LPT assigns each of the jobs to the individual machine *m* starting with the one having the least workload. The LPT determines load per machine, which is denoted as *ml* using the expression given in Eq (1):

$${ml}_{k}=\frac{1}{m-1}\left(\sum_{j=1}^{n}\left(\underset{\begin{array}{c} k=1,2,\ldots ,m\\ i=1,2,\ldots ,n \end{array}}{\min}\left(P_{j,k}+S_{i,j,k}\right)\right)\right)$$ (1)

where the expression ∑_*i*≠*j*_(*p*_*j*.*k*_ + min_*k*_ *S*_*i*,*j*,*k*_) represents the total work content of all jobs in the problem and *k* is the machine index. In addition, the proposed LPT model used in this paper ensures that those jobs with processing times are placed more towards the end of the generated schedule so as to maintain load balancing on all machines.

### Symbiotic organisms search algorithm

The SOS algorithm is a population based metaheuristic algorithm introduced by Cheng and Prayogo in 2014 [42]. The algorithm was first developed for solving numerical engineering optimization problems on continuous real space. It replicates the symbiotic relationship interaction existing amongst organisms in the ecosystem. The symbiotic interaction strategy is often adopted by organisms for their survival and modeled in the physical sense to find the fittest organisms in the solution search space. Like most population based metaheuristic algorithms, SOS possesses a number of interesting features, some of which include using candidate solutions from a population of organisms over a search space to find the global solution [3]. Its search processes are guided by candidate solutions based on some special operators. The quality of solutions is preserved using a specific selection mechanism requiring the proper setting of some common control parameters for its operation. However, as mentioned in the introductory section, the SOS differs from other population-based algorithms in that it uses few control parameters and requires no parameter fine-tuning. These characteristics are considered as the advantages which the SOS have over other similar algorithms.

The implementation process of the algorithm commences with the creation of an initial population of ecosystem matrix, with each row of organism being considered as a candidate solution to the corresponding problem. The search process begins after the initialization stage is completed, following the generation of an initial ecosystem population. The new candidate solutions are evaluated by simulating the continuous interactions between two organisms in the ecosystem using three symbiotic interaction phases namely, mutualism, commensalism, and parasitism. The mutualism phase involves an interaction where an organism engages in a relationship that benefits all parties involved. In the commensalism phase, an individual organism develops a relationship that attracts benefits to itself alone, while the other organism is left unharmed. However, in the parasitism phase, the developed relationship only benefits one organism and harms the other. These evaluation phases employed by the algorithm is adopted by the individual organisms and used to increase their fitness and survival advantages [42]. The evaluation and updating of the best organism during the search mechanism is an iterative process which is performed until the termination condition is met. The computational procedures for the standard SOS are illustrated as shown in the Algorithm listing 1. The SOS algorithm uses two control parameters, namely *ecosize* and *maxIt*. The parameter denoted as *ecosize* represents the number of organisms in the ecosystem, which is usually called the population size. The parameter *maxIt* is the maximum number of iteration. A detailed description and formulation of the three algorithm phases are presented in the algorithm scheduling procedure section below (Algorithm 1).

**Algorithm 1**: Standard SOS procedure

1: Setup control parameter: initial ecosystem, population size: *ecosize*, maximum number of iteration: *maxIt*

2: ***While***(*termCondition* < *maxIt*) // *termCondition* is the user defined termination condition

3:         ***For*** *counter* = 1 to *ecosize*

4:                 Determine the best organism

5:                 Mutualism Phase

6:                 Commensalism Phase

7:                 Parasitism Phase

8;         ***End for***

9:         Print out global best solution

10: ***End While***

## Scheduling heuristic components

This section describes the various components of the proposed SOS algorithms. Following on the description of the basic SOS algorithm presented in scheduling heuristics section, some new features and modifications have been introduced into the basic SOS to make it more suitable to handle the scheduling of the parallel machines. The five essential components used for the improvement of the proposed SOS method, namely, solution representation, initial solution, SOS update phase, SOS algorithm procedure and local search improvement, with detailed descriptions following.

### Solution representation

The design of an appropriate encoding scheme increases the effectiveness of the SOS algorithm to handle the one-to-one mapping between candidate solutions and individual organisms. For the problem at hand, a similar representation pattern presented in [14, 16] is adopted, where the solution representation of assigning *n* jobs to *m* machines is represented as a vector *S*_1_ whose dimension is equal to the number of jobs. Assuming there are 16 jobs (*n* = 16) and 4 machines (*m* = 4), then the following vector *S*_1_ = [4,1,4,4,4,1,3,3,3,3,2,2,2,2,1,1] implies that the first machine *m*_1_ will be assigned the following jobs: *m*_1_ = {2,6,15,16}, the second machine *m*_2_ will be assigned the following jobs: *m*_2_ = {11,12,13,14}, the third machine *m*_3_ will be assigned the following jobs: *m*_3_ = {7,8,9,10}, and the fourth machine *m*_1_ will be assigned the following jobs: *m*_4_ = {1,3,4,5}. For clarity’s sake, the solution representation of the machine assignment vector is shown in Table 1.

**Table 1:** Illustration of machine assignment vector.

*job*_*i*_| *i* = 1,2,..*n*	1	2	3	4	5	6	7	8	9	10	11	12	13	14	15	16
*m*_*k*_| *k* = 1,2,…*m*	4	1	4	4	4	1	3	3	3	3	2	2	2	2	1	1
	*m*_4_	*m*_1_	*m*_4_	*m*_4_	*m*_4_	*m*_1_	*m*_3_	*m*_3_	*m*_3_	*m*_3_	*m*_2_	*m*_2_	*m*_2_	*m*_2_	*m*_1_	*m*_1_

Therefore, the operation sequence denoted in this case can be represented by *S*_2_ as *m* × *n* matrix that shows the sequence of operations on each machine. Consider the following instance of *S*_2_ given as

$$S_{2}=\left[\begin{array}{cccccccccccccccc} 6 & 15 & 16 & 2 & 0 & 0 & 0 & 0 & 0 & 0 & 0 & 0 & 0 & 0 & 0 & 0\\ 12 & 11 & 13 & 14 & 0 & 0 & 0 & 0 & 0 & 0 & 0 & 0 & 0 & 0 & 0 & 0\\ 8 & 9 & 10 & 7 & 0 & 0 & 0 & 0 & 0 & 0 & 0 & 0 & 0 & 0 & 0 & 0\\ 3 & 4 & 5 & 1 & 0 & 0 & 0 & 0 & 0 & 0 & 0 & 0 & 0 & 0 & 0 & 0 \end{array}\right]$$

The variable *S*_1_ or *S*_2_ describes the sequence of operation for each machine, while the sequence of operation in machine *m*_1_ is job 6, job 15, job 16, and job 2. The same description applies to machines *m*_2_ and *m*_3_. The zeros after job 2, job 14, job 7, and job 1 indicate that these jobs are the last to be processed by *m*_1_ for job 2, *m*_2_ for job 14, *m*_3_ for job 7 and *m*_4_ for job 1.

In the SOS algorithm, since machine assignment is performed by the LPT heuristic, each organism carries only the information of job sequencing, such as the favorability of sequencing job *j* after job *i* in machine *k*. It is important to note that machine *k* is only assigned a job when its total workload is less than that of machine *l*, that is following the LPT dynamic load balancing condition. In the decoding process for the organism, jobs are selected one by one from the set of unscheduled jobs and assigned processing machines according to the described LPT procedure until all the jobs are scheduled, after which the total completion time corresponding to the organism is calculated and then used to evaluate the performance of each organism as described in the algorithm update phase section.

### Initial solution

The initial solution is constructed by assigning all jobs to the available sets of processing machines. The initial solution is randomly generated using the design variables which are the unscheduled jobs and *m* machines. The randomly generated solution is considered as the initial ecosystem which comprises of individual organisms that correspond to the choice of job sequencing operation *S*_2_ on a specific selected machine encoded in a matrix of *m* × *n* dimension as described in algorithm solution representation section above, where *m* and *n* denote the number of machines and jobs. Let us assume that *X*_*k*_ is the *k*th position of the organism in the solution search space, then *X*_*k*_(*j*) denotes the machine where job *j* is assigned by the LPT heuristic. The position of the organism is updated through the iterative phases of the SOS procedure described in the algorithm update phase section.

### Algorithm update phase

The update variable denoted by *X*_*best*_ in the first two update phases (mutualism and commensalism) of the algorithm stores the best organism position, which is evaluated based on its corresponding objective value (makespan). The organism’s objective value is obtained by the decoding process that incorporates the LPT machine assignment rule. The update parameter *X*_*best*_ is usually updated for each *X*_*k*_(1), *X*_*k*_(2), …, *X*_*k*_(*n*), after which the organism with overall *X*_*k*_(*j*) best is saved as the global best cost. The details of the proposed SOS algorithm procedure are presented next.

### Algorithm scheduling procedure

In describing the proposed algorithm’s strategy for generating sequencing solutions for *S*_1_ and *S*_2_, it is noteworthy to mention that the novel aspect of this solution approach is the use of two heuristic techniques, LPT and SOS, in solving the parallel machine scheduling problem. One technique is used for assigning jobs to machines, which is the LPT heuristic. The other technique is the new SOS metaheuristic algorithm used for generating solutions *S*_1_ and *S*_2_ by optimizing the sequencing of the assigned jobs in each machine. Prior to the search process, the algorithm starts with control parameter setups, followed by the random generation of the initial ecosystem population of *S*_1_ or *S*_2_. An organism *X*_*j*_ with the sequencing operation information is randomly selected from the population and its corresponding objective value (*C*_*max*_), which is computed using the LPT heuristic evaluated against one of the organisms *X*_*i*_ in the initial ecosystem, as explained in the initial solution section. The organism with the current best sequencing operation solutions (*S*_1_ or *S*_2_) and best objective value (*C*_*max*_), is set to be the best organism (*X*_*best*_). The SOS search process begins immediately after the initialization stage, by iteratively updating each organism in the ecosystem as explained above in the algorithm update phase section, while the organism benefits from the continuous interaction with other organisms in the population based on the following three interaction phases:

- ***Mutualism phase***: in this phase the organism *X*_*j*_ is randomly selected from the ecosystem to mutually interact with the organism *X*_*i*_ (where *X*_*i*_ ≠ *X*_*j*_), with the sole aim of increasing their mutual survival advantage in the ecosystem. The resulting solutions, *X*_*inew*_ and *X*_*jnew*_, that are the consequences of this interaction, are calculated based on Eqs (2) and (3)
  $$X_{inew}=X_{i}+r_{1}(X_{best}-\varphi \times f_{1})$$ (2)
  $$X_{jnew}=X_{j}+r_{1}(X_{best}-X_{mutual}\times f_{2})$$ (3)
  where
  $$\varphi =\frac{X_{i}+X_{j}}{2}$$ (4)
  *r*_1_ and *r*_2_ are uniformly distributed random numbers in the range of [0, 1]. The term *φ* in Eq (2) and defined in Eq (4), is known as the *MutualVector* and it represents the relationship between the two organisms *X*_*i*_ and *X*_*j*_. The term *X*_*best*_ denotes the highest degree of adaptation for the organisms. The terms *f*_1_ and *f*_2_ denote the mutual benefit factors, which represent the level of benefit that both *X*_*i*_ and *X*_*j*_ can derive from the mutual association, since either of the organisms can get a partial or full benefit from the interaction, both *f*_1_ and *f*_2_ are determined by randomly using the values 1 or 2. The values 1 and 2 denote partial and full benefits, respectively. The new candidate solutions, *X*_*inew*_ and *X*_*jnew*_, are however, only accepted if they give better fitness values than the previous solutions.
- ***Commensalism phase***:Similar to the mutualism phase, an organism *X*_*j*_ is randomly selected from the ecosystem’s population and made to interact with the organism *X*_*i*_. The relationship interaction is such that only one organism benefits from the interaction. For example, the organism *X*_*i*_ derives benefit from its interaction with *X*_*j*_, while *X*_*j*_ does not benefit and neither is it harmed as a result of the interaction. The new organism is updated as shown in Eq (5).
  $$X_{inew}=X_{i}+r_{1}(X_{best}-X_{j})$$ (5)
  where the term *X*_*best*_ – *X*_*j*_ represents the benefit provided by the organism *X*_*j*_ to assist *X*_*i*_ increase its level of survival advantage in the ecosystem.
- **Parasitism phase:** In this phase, an artificial parasite vector denoted by *X*_*pv*_ is created in the problem search space by mutating the organism *X*_*i*_ and then modifying its randomly selected dimensions using a random number. The organism *X*_*j*|*i*≠*j*_ is selected randomly from the ecosystem’s population to serve as a host to the *X*_*pv*_. The evaluation is carried out in such a way that, if the fitness value of the *X*_*pv*_ is better than that of the organism *X*_*j*_, then *X*_*pv*_ will replace the position of *X*_*j*_ in the population, otherwise, if the fitness value of *X*_*j*_ is better, then *X*_*j*_ will build an immunity against *X*_*pv*_, after which *X*_*pv*_ is removed from the list of the population.

The standard SOS algorithm was originally implemented to work on a continuous domain. However, the problem described in this paper is essentially an optimization problem that involves the discrete search domain (which is an integer space of alignment of job indices). Therefore, to obtain a corresponding discrete solution for the ***P***_***m***_**|*S***_***i*,*j*,*k***_**|*C***_***max***_ optimization problem with integer variables, the round function in MATLAB is employed to convert the resulting floating variable solutions to the nearest integer. Thus the previous SOS formulated Eqs in (2), (3), and (5) are then transformed as follows:

- mutualism
  $$X_{inew}=round\{X_{i}+r_{1}(X_{best}-\varphi \times f_{1})\}$$ (6)
  $$X_{jnew}=round\{X_{j}+r_{2}(X_{best}-\varphi \times f_{2})\}$$ (7)
- commensalism
  $$X_{inew}=round\{X_{i}+r_{1}(X_{best}-X_{j})\}$$ (8)

The pseudocode of the SOS algorithm procedures described above is as presented in Algorithm 2 below.

**Algorithm 2**: Standard SOS-LPT pseudocode

1: Using LPT rule, create initial schedule as *X =* {*X*_1_,*X*_2_,…,*ecosize*} for SOS and evaluate its fitness

2: Solve assignment task using the LPT heuristic to find *S*_1_ according to Eq (1)

3: Solve sequencing tasks by using SOS to find *S*_2_

4: Identify the best solution of the initial schedule *X*_*best*_ (*C*_*max*_) that is associated with *S*_1_ and *S*_2_

5:         ***for*** *it* = *maxIt*

6:             ***for*** *i* = 1 *to ecosize*

7:             //**Mutualism Phase**

8:                 Randomly select *X*_*j*_, where i ≠ j

9:                 *f*_1_ ← (1 + *round*(*rand*(0,1))

10:                 *f*_2_ ← (1 + *round*(*rand*(0,1))

11                 *r*_1_ ← *rand*(0,1)

12                 *r*_2_ ← *rand*(0,1)

13:                 *φ* ← (*X*_*i*_ + *X*_*j*_)/2

14:             ***for*** *k* = 1 to *n*//*n* is the problem dimension (or number of jobs)

15:                     *X*_*inew*_[*k*] ← *round*{*X*_*i*_[*k*] + *r*_1_ × (*X*_*best*_[*k*] − *φ*[*k*] × *f*_1_)}

16:                     *X*_*jnew*_[*k*] ← *round*{*X*_*j*_[*k*] + *r*_2_ × (*X*_*best*_[*k*] − *φ*[*k*] × *f*_2_)}

17:                 ***if*** *f*(*X*_*inew*_) < *f*(*X*_*i*_) ***Then***

18:                     *X*_*i*_ ← *X*_*inew*_

19:                 ***end if***

20:                 ***if*** *f*(*X*_*jnew*_) < *f*(*X*_*j*_)

21:                     *X*_*j*_ ← *X*_*jnew*_

22:                 ***end if***

23:             //**Commensalism Phase**

24:                 Randomly select *X*_*j*_, where i ≠ j

25:                 *X*_*inew*_[*k*] ← *round*{*X*_*i*_[*k*] + *r*_1_ × (*X*_*best*_[*k*] − *X*_*j*_[*k*])}

26:                 ***if*** *f*(*X*_*inew*_) < *f*(*X*_*i*_) ***Then***

27:                     *X*_*i*_ ← *X*_*inew*_

28:                 ***end if***

29:             //**Parasitism Phase**

30:                 Randomly select *X*_*j*_, where i ≠ j

31                   Create a parasite vector *X*_*pv*_ from *X*_*i*_

32:                 *X*_*pv*_[*k*] ← *X*_*i*_[*k*]

33:                 ***if*** *f*(*X*_*pv*_) < *f*(*X*_*j*_) ***Then***

34:                     *X*_*j*_ ← *X*_*pv*_

35:                 ***end if***

36             ***end for***

37:                 Update the current *X*_*best*_ using the iterated local search mechanism (Algorithm 3)

38:             ***end for***

39:         ***end for***

The SOS-LPT algorithm follows through each of the steps highlighted in Algorithm 2, starting with the initialization of the ecosystem *X*_*i*_ of size *ecosize*. The *ecosize* which denotes the population of the organisms is usually set within a relatively small value of *ecosize* (≤ 25), a relatively moderate vale of *ecosize* (≤ 50) and a relatively large value of *ecosize* (≤ 100). However, the size of the population can also be defined, depending on the objective function of the problem at hand. The reason is there are instances when selecting a small population size yields a better result than a large population size and vice versa. After the initialization process, the algorithm creates and evaluates each new organism’s position by computing and comparing their respective objective function values in such a way that the organism with the best objective value is selected as *X*_*best*_. Iteratively, the process is repeated by updating the current solution with the best solution ever found, until the organism with the global best solution is discovered. The algorithm execution is terminated when the maximum iterations criterion is attained or the fitness evaluation is met. Otherwise, the algorithm continues to evaluate by exploring and exploiting other new possible solution search spaces. However, the stopping condition denoted by *maxIt* is quite an important factor that can determine the final result of the simulation. For example, if the algorithm is stopped too early, the approximation of the solution might not be close to the targeted global optimum and prolonging the simulation might as well incur unnecessary scale up in the computational effort.

The integration of a local search strategy into the SOS algorithm has proven to be efficient in providing very competitive solutions [43, 44]. Therefore, an iterated local search strategy has been included in the implementation of the SOS algorithm for the current problem. The local search improvement step which is explained below is applied after the end of the movement in each phase of the algorithm evaluation, on the condition that *X*_*best*_ does not improve after each round of the evaluation process. The SOS uses the local search to generate neighboring solutions for *S*_1_ and *S*_2_ for each organism for which the neighboring solution’s *C*_*max*_ is compared with the generated solution’s *C*_*max*_ (*S*_1_ or *S*_2_). If the solution generated by the local search after each iteration is better, then the local search solution is used to update each organism. The neighboring solutions for *S*_1_ is generated by reversing the initial machine assignment generated by the LPT heuristic for the available *n* jobs, while the neighboring solution for *S*_2_ is generated by swapping two randomly generated jobs in the *n* × *m* dimension matrix. The insertion operation was also applied where necessary to generate solutions for either *S*_1_ or *S*_2_. The discussion on each of these operators is presented in the following section.

### Local search

An iterated local search method is implemented and applied to improve the local search phase of the proposed algorithm. The application of iterated local search to parallel machine scheduling is not new, and interested readers are referred to [1, 45] for more information in this regard. The term ‘improvement’ refers here to a reduction in the current solution’s total completion time (*C*_*max*_). The iterated local search procedure employed here consists of the application of a series of random insertion and swap moves to the local optimum solution generated by the LPT rule. An insertion move removes a job *i* from machine *k* and inserts it into another machine *l*, the swap move selects two jobs *i* and *j* and exchange their machine assignment, while the reversion move replaces a randomly selected job’s sub-assignment by its reversal. The procedures used for applying the set of combined random insertion and swap moves, are as presented in the kick move Algorithm listing 3 (Algorithm 3).

**Algorithm 3**: Pseudocode for the iterated and the local search random moves (kick move)

***Function*** kickMove (X)

1: ***While*** (*π*) ***do //*** *π* denotes the number of moves

2:         Randomly select two machines *k* and *l* such that *k* ≠ *l*

3:         Randomly select two jobs *m*_*k*_(*i*) and *m*_*l*_(*j*)

4:         Apply(*Swap*(*m*_*k*_(*i*),*m*_*l*_(*j*)))

5:         Apply(*Reverse*(*m*_*k*_(*i*),*m*_*l*_(*j*)))

6:         Apply(*Insert*(*m*_*k*_(*i*),*m*_*l*_(*j*)))

7: ***End while***

In Algorithm 3, the value of *π* (number of random moves) depends on the number of machines. In this experiment, the values of *π* between the intervals of [0.5m, 0.9m], were carefully selected to fine-tune the algorithm. The computational complexity of the proposed LPT heuristic and SOS algorithm can be calculated as follows: the cost of obtaining the initial scheduling and computing the corresponding jobs completion times by the LPT can be determined in *O*(*n log n*) times. The cost of determining the total load per machines and makespan can be calculated in constant time, which can be determined in *O*(1) times. The average-case time complexity of the kick move operation is *O*(*n*^2^), while the total time complexity of the proposed algorithm is *O*(*n*^2^).

## Experimental setup and results

In this section, the performance of the two developed algorithms, SOS and SOS-LPT, are analyzed with other methods from the literature. Both algorithms were implemented in MATLAB R2015a and run on Intel ® Core™ i3 M300 @ 2.13 GHz with 4GB memory running under Windows 7, 64 bits. The computational tests conducted were divided into three experiments. In the first experiment, the researchers compared the performance of the SOS with the PSA. The algorithms were tested using randomly generated test instances. The parameter setting for the five algorithms considered in this paper are as described in Table 2. In the second experiment, the numerical results obtained were compared by running SOS and SOS-LPT on 15 replication of existing benchmark problem instances available at [38].

**Table 2:** Parameter settings for the five algorithms.

PSA	ACOII	GADP2	SADP	SOS
***NP* = 100**	*NP* = 50	*NP* = 100	*T*_0_ = *LB* + (*UB* − *LB*)/10	*NP* = 50,100
***T***_**0**_ **= 10**	*ρ* = 0.01	*pc* = 0.6	*T*_*f*_ = *LB*	*π* = [0.5*m*,0.9*m*]
***T***_***f***_ **= 0.001**	*φ* = 0.081	*pm* = 0.5	*α* = 0.99	
***α* = 0.99**	*τ* = 10			
	*τ*′ = 10			
	*LocalIter* = 50			

Note: NP = population size or eco size or number of ants; *T*_0_ = initial temperature; *T*_*f*_ = final temperature; temperature reduction rate = *α*; pheromone evaporation = *ρ*; global update rates = *φ*; pheromone amounts = *τ*; local search iteration = *LocalIter*; *pc* = crossover rate; *pm* = mutation rate; *π* = number of random move; m = number of machines.

In the third experiment, the numerical results of both SOS and SOS-LPT were compared against other existing techniques from the literature. The processing time and setup times, which were obtained from [38] follows a randomly generated uniform distribution on the interval [50,100]. For fair comparison the two main methods proposed in this paper, namely SOS and SOS-LPT with the other compared algorithms, that is ACO with local search (ACOII), hybrid genetic algorithm with dominance properties (GADP2) heuristic [27], and hybrid simulated annealing with dominance properties (SADP) heuristic [27]), are executed under the same experimental conditions using the same benchmark data available at [38]. The obtained results are analyzed by using the percentage deviation (PD) from the lower bound (LB), which was calculated as follows:

$${PD}_{LB}=\frac{C_{max\_algorithm}-LB}{LB}\times 100\%$$ (9)

While the PD from SOS was calculated as follows:

$${PD}_{SOS}=\frac{C_{max\_algorithm}-C_{max\_SOS}}{C_{max\_SOS}}\times 100\%$$ (10)

where *C*_max_*algorithm*_ and *C*_max_*SOS*_ are the solutions obtained by the other algorithms and SOS, respectively, for the fifteen (15) replicate runs carried out for each method. Similarly, the formula in Eq (10) was also used to calculate the percentage deviation from SOS-LPT (*PD*_*SOS*–*LPT*_). The LB used in this paper was adopted based on the model from [45] and is presented as follows:

$$LB1=\frac{1}{m}\sum_{j=1}^{n}\underset{\begin{array}{c} k=1,2,\ldots ,m\\ i=1,2,\ldots ,n \end{array}}{\min}\left[P_{j,k}+S_{i,j,k}\right]$$ (11)

$$LB2=\underset{j=1,2,\ldots ,n}{\max}\{\underset{\begin{array}{c} k=1,2,\ldots ,m\\ i=1,2,\ldots ,n \end{array}}{\min}\left[P_{j,k}+S_{i,j,k}\right]$$ (12)

LB=max⁡(LB1,LB2) (13)

It is noteworthy to mention that the three set of existing algorithms selected for comparison with the proposed techniques were chosen on the basis that the same type of balanced machine benchmark datasets was also used for their evaluation.

### First experiment

Table 3 presents the results of the first experiment conducted in this study, which is the evaluation between SOS and PSA. The first and second columns of this table show the sizes of the test instances for machines and jobs. The third “C_max_” and fourth “times” columns represent the average makespan and CPU run times in seconds for the 20 problem instances tested respectively. The comparison is carried out in this form to firstly test the performance of the SOS algorithm over a wide range of randomly generated datasets. Furthermore, a PSA was implemented and used to evaluate the efficiency of the SOS. Interested readers could consult [46] for more details on the standard SA implementation for solving the UPMSP problem. Therefore, since the two algorithms, SOS and PSA, are population based methods, the comparison can be justified by using the same platform, datasets and computational environment. The processing time, which was randomly generated followed a discrete uniform distribution (DUD) on (10,100) and the setup time followed a DUD on (1,10). The values of jobs and machines were varied. For the jobs instance, the selected values were *n* = 10,20,30,50,100, while for the machines instance, the selected numbers were *m* = 2,3,4,6,8,10. The number of random moves or kick moves (*π*) used for the SOS implementation was set at interval [0.5m, 0.9m].

**Table 3:** Comparison between SOS and SA, with SOS as the reference algorithm.

Machines	Jobs	PSA		SOS		Improvement
		*C*_*max*_	Time(s)	*C*_*max*_	Times(s)	*PD*_*SOS*_(%)
2	10	207	3.95	196	4.71	5.31%
4	20	254	13.98	138	7.59	45.67%
2	30	879	21.62	754	10.58	14.22%
3	30	420	18.9	272	10.6	35.24%
4	30	427	22.32	292	10.6	31.62%
6	30	307	26.18	145	10.62	52.77%
8	30	273	26.96	95	10.68	65.20%
10	30	222	36.34	77	10.75	65.32%
2	50	1419	33.09	1137	16.34	19.87%
3	50	929	44.4	614	16.49	33.91%
4	50	764	44.92	419	16.56	45.16%
6	50	558	66.91	219	16.45	60.75%
8	50	463	69.02	157	16.6	66.09%
10	50	407	71.07	127	16.85	68.80%
2	100	2697	130.67	2077	31.35	22.99%
3	100	2002	131.28	1236	31.43	38.26%
4	100	1633	133.42	820	31.67	49.79%
6	100	1092	134.35	482	31.67	55.86%
8	100	954	155.39	324	33.7	66.04%
10	100	809	197.19	253	31.66	68.73%

From the results presented in Table 3, it can be observed that SOS performed better than PSA as regards solution quality and average CPU time. Similarly, SOS outperformed PSA in all cases for the varying problem instances tested in terms of solution quality, because of the iterated local search improvement mechanism incorporated into the SOS algorithm. Since the main goal of the improvement strategy is to bring about a reduction in the current solution’s total completion time, it is not surprising that the SOS has gained tremendously over the PSA with respect to the average CPU execution time of the two algorithms. As regards the overall performance of the two methods, the results in Table 3 show that the relative performance of SOS and PSA increases with increase in the problem size.

### Second experiment

In the second experiment, the two developed algorithms were tested on a total of 450 out of the 540 problem instances combination. The available balanced machine benchmark dataset in [38] has 15 replications of problem combination comprising of 20, 40, 60, 80, 100 and 120 numbers of jobs. The number of machines in this combination includes 2, 4, 6, 8 and 10. In this experiment, only up to 10 machines were considered. By referring to balanced machine dataset, it is meant that the data distribution for processing time (*P*_*i*,*k*_) and setup times (*S*_*i*,*j*,*k*_) is balanced. In Table 4, results of all the tests instances are shown for the evaluated algorithms. This table presents the minimum, average, and maximum makespan simulation results for SOS and SOS-LPT. The gap between these two methods is also computed in the form of percentage deviation using the formula described in Eq (11).

**Table 4:** Comparison between SOS and SOS-LPT algorithms, with SOS-LPT as the reference algorithm.

Machines	Jobs	SOS (1)				SOS-LPT (2)				Improvement
		Min	Avg	Max	StDev	Min	Avg	Max	StDev	*PD*_*SOS*–*LPT*_(%)
2	20	1152	1224.12	1260	30.48	1152	1200.86	1236	20.80	1.9369
	40	2363	2377.86	2393	9.52	2360	2376.27	2393	10.60	0.0669
	60	3519	3565.61	3631	32.86	3519	3556.66	3631	26.34	0.2516
	80	4705	4734.86	4754	11.44	4705	4725.20	4754	13.85	0.2044
	100	5789	5838.87	5976	65.07	5793	5829.40	5976	49.11	0.1625
	120	6996	7050.04	7238	70.20	6881	7021.53	7247	129.42	0.406
4	20	541	592.26	643	8.33	539	563.33	601	6.01	2.9562
	40	1085	1132.13	1183	28.89	1076	1129.34	1183	33.48	0.247
	60	1684	1718.68	1752	23.81	1684	1705.72	1752	16.42	0.7598
	80	2256	2296.52	2320	18.51	2256	2284.53	2320	18.60	0.5248
	100	2808	2840.84	2903	20.90	2778	2821.07	2915	41.58	0.7008
	120	3380	3404.74	3449	22.68	3331	3397.06	3446	43.01	0.2261
6	20	380	386.26	393	3.77	381	386.21	393	3.78	0.0129
	40	734	750.47	763	9.08	734	741.28	750	4.01	1.2397
	60	1104	1118.05	1167	16.04	1096	1106.01	1113	5.56	1.0886
	80	1500	1506.46	1514	4.91	1491	1502.92	1514	6.87	0.2355
	100	1868	1879.93	1893	8.46	1868	1874.23	1892	6.21	0.3041
	120	2245	2255.86	2266	6.75	2239	2244.47	2266	8.42	0.5074
8	20	276	282.18	291	4.26	270	275.87	286	3.91	2.2873
	40	524	561.37	601	4.51	522	538.39	599	4.15	0.3631
	60	819	832.93	845	8.61	803	822.24	840	10.98	1.3001
	80	1092	1116.66	1134	14.25	1046	1092.17	1134	27.68	2.2423
	100	1367	1378.30	1390	8.77	1341	1361.42	1390	16.80	1.2399
	120	1673	1681.68	1690	4.73	1643	1659.66	1690	16.09	1.3268
10	20	177	231.12	262	2.10	167	225.17	281	5.07	3.9461
	40	409	447.48	489	5.63	394	425.86	446	9.31	3.8368
	60	641	648.15	655	4.71	602	632.34	655	17.11	5.1473
	80	866	874.40	884	5.99	842	862.33	884	13.32	1.3997
	100	1111	1121.53	1127	5.34	1029	1077.6	1127	34.43	4.0767
	120	1324	1335.31	1355	10.36	1290	1314.26	1355	17.96	1.6017

**Fig 2:**
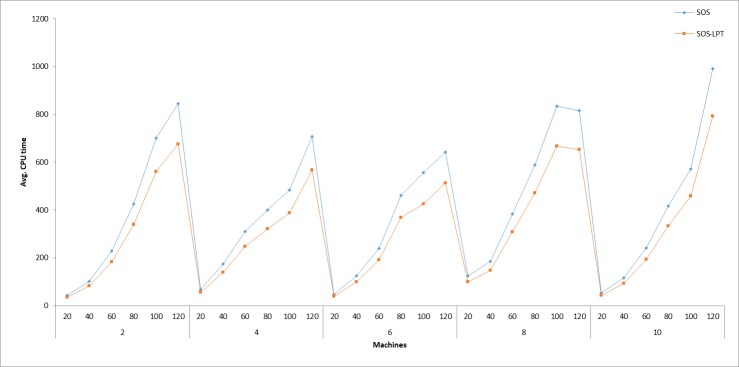
Comparison of SOS and SOS-LPT average CPU execution time.

The results in Table 4 show that the performance of the SOS-LPT algorithm is better than that of the SOS in both quality of solution and average CPU time. For the average CPU time illustrated in Fig 2, the excellent performance of the SOS-LPT can be explained that by incorporating LPT assignment heuristic into the SOS, the SOS-LPT algorithm needed to only perform its search on the different job sequencing solutions generated by the LPT, unlike in the case of the SOS, where the search space, even though efficient, still lacks such additional refinement and focused search direction introduced by the incorporation of the LPT heuristic. Therefore, it can be concluded that the LPT assists to speed up the search process for the SOS algorithm by reducing the solution search space. In addition, a noticeable increase in the solution quality of the SOS was observed, indicating that LPT also improves the performance of the SOS in finding better solution, *C*_*max*_.

In this paper, only the SOS’s and SOS-LPT’s average CPU times for all the different problem structures described above, are reported. The average CPU times were obtained by running 15 replicates of each problem instance for 500 iterations. From Fig 2, it can be noticed that as the number of jobs increased, the computational time for both the SOS and SOS-LPT also increased. Increasing the number of machines likewise increased the algorithm running times, as it was observed with the instance of running 20 jobs on 2 machines, where the average CPU times of 43.10 seconds and 34.48 seconds were recorded for SOS and SOS-LPT, as compared to the 844.61 seconds and 674.68 seconds for running 20 jobs on 120 machines. The main reason for introducing the LPT heuristic into the SOS as aforementioned was to minimize the duration of each problem execution time. The results of the CPU times illustrated in Fig 2, show that this goal has been achieved. For example, the average CPU time for the SOS-LPT to solve 120 jobs on 10 machines, is 792.51 seconds compared to the SOS’s 990.64 seconds, which makes SOS-LPT a more suitable candidate algorithm in terms of speed to be used in real-world practice.

### Third experiment

In the third experiment, SOS and SOS-LPT are compared with other methods from the literature, namely ACO [16], ACOII [14], GADP2 and SADP [27]. The problem instance combination for the comparison with ACO and ACOII included 20, 40, 60, 80 100, and 120 jobs, while the machine instances included 2, 4, 6, 8, 10, and 12 number of machines. In summary, the problem set used for this experiment consists of 15 instances for each combination of machine number, job number, processing time distribution, and setup time distribution, resulting in a total of 1,620 (6 × 6 × 3 × 15) test instances. However, for the hybrid SADP and GADP2, the problem combination included 20, 40, 60, and 80 jobs, while 2 and 6 machines were considered. The ACO algorithm considered is a hybrid of the classical ACO and local search implementation, while the ACOII is an extended version of the ACO. The GADP2 is a hybrid of genetic algorithm and dominance properties heuristic and SADP is a hybrid of simulated annealing and dominance properties heuristic. Therefore, to justify the rationale behind selecting these algorithms for the purpose of evaluation, the algorithms hybridization structures were compared with the researchers’ proposed methods. It is reasonable therefore, to deduce that the ACO can be compared with SOS implementation, which is a mix of basic SOS and iterated local search strategy. Similarly, the dominance properties heuristics (DP) of GADP2 and SADP can be compared with the SOS with LPT heuristic.

**Fig 3:**
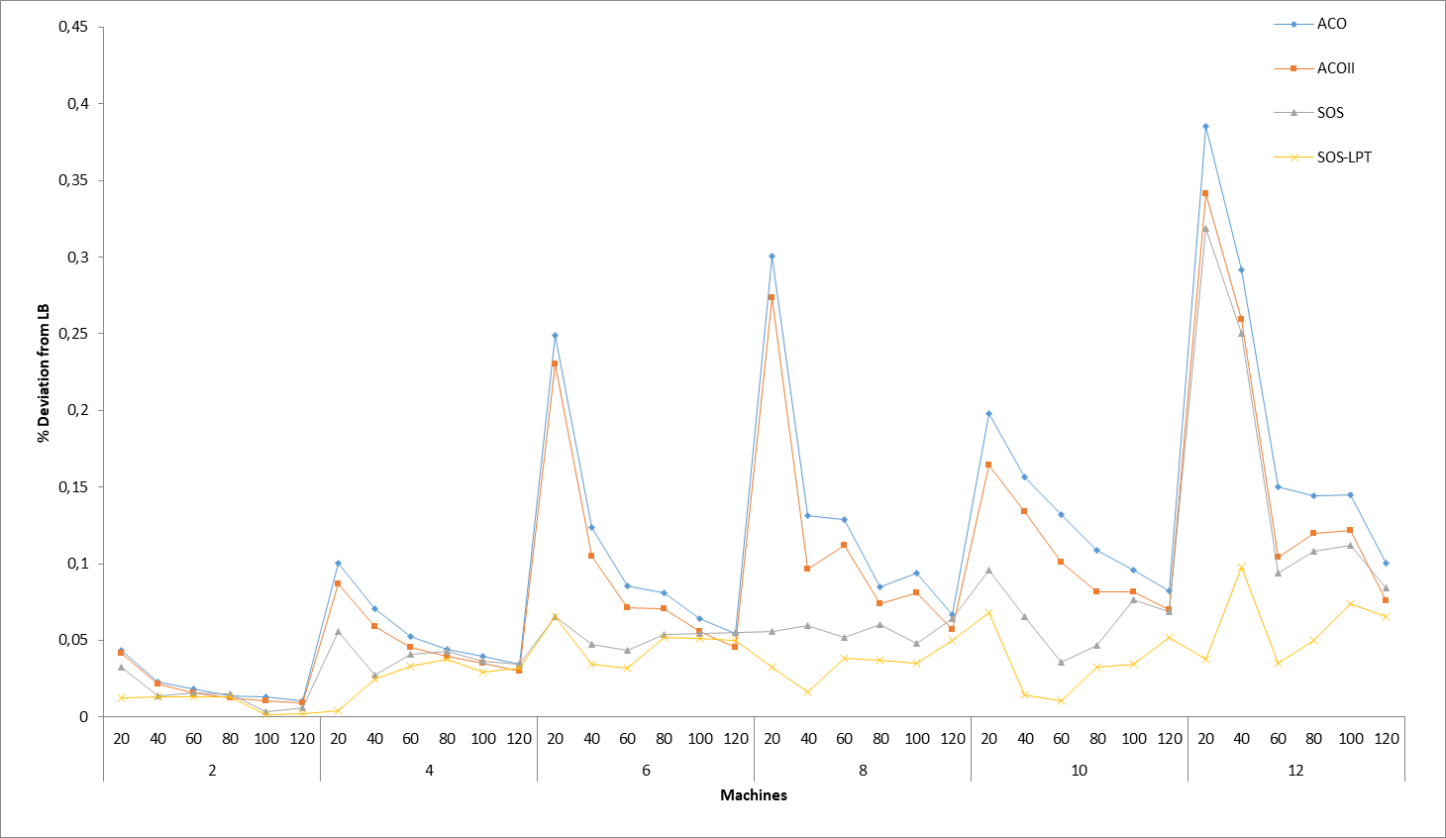
Percentage deviation of each algorithm with LB as the control algorithm.

In Tables 5 and 6, results of all the tests instances are shown for the evaluated algorithms. However, only the results obtained by SOS and SOS-LPT originated from the researchers’ tests computation, while the results of other heuristics were directly obtained from [38], which contains the sets of all benchmark problem instances with their respective solutions that were also used for this implementation. Table 6 presents the minimum, average, and average makespan results for the compared algorithms. As indicated in Table 5, ACO and ACOII are outperformed by both SOS and SOS-LPT, with SOS-LPT having the best results, and closely followed by the SOS. Similarly, in Table 6 the performances of the SADP and GADP2 were outperformed by SOS and SOS-LPT, still with SOS-LPT emerging the overall best performed algorithm in all the problem instances tested. The least performed method is the SADP algorithm, since it has the highest average *C*_max_ values for most of the tests results carried out. Fig 3 summarizes the relative percentage deviation of ACO, ACOII, SOS, and SOS-LPT from the LB for all the problem combination considered.

**Table 5:** Experimental results for 15 replicated runs of ACO, ACOII, SOS, and SOS-LPT algorithms.

Machines	Jobs	LB	ACO	ACOII	SOS	SOS-LPT
**2**	20	1185.833	1237.8	1235.267	1224.12	1200.86
	40	2344.7	2397.8	2394.933	2377.86	2376.27
	60	3510.167	3574.6	3565.133	3565.61	3556.66
	80	4664.833	4730.4	4722.867	4734.86	4725.2
	100	5819.233	5897.6	5881.933	5838.87	5829.4
	120	7008.033	7082.6	7072.667	7050.04	7021.53
**4**	20	560.8333	617.1333	609.4667	592.26	563.33
	40	1101.883	1179.867	1166.933	1132.13	1129.34
	60	1650.733	1737.933	1725.667	1718.68	1705.72
	80	2201.483	2298.533	2288.933	2296.52	2284.53
	100	2740.7	2849.933	2837.8	2840.84	2821.07
	120	3291.2	3405.133	3389.867	3404.74	3397.06
**6**	20	362.3999	452.7333	445.8667	386.26	386.21
	40	716.5556	805.4	791.9333	750.47	741.28
	60	1071.478	1163.467	1147.8	1118.05	1106.01
	80	1429.121	1545.333	1530.467	1506.46	1502.92
	100	1783.033	1897.467	1882.467	1879.93	1874.23
	120	2137.599	2253.933	2234.2	2255.86	2244.47
**8**	20	267.225	347.6	340.2667	282.18	275.87
	40	529.7583	599.2667	580.7333	561.37	538.39
	60	791.7417	893.8	880.4667	832.93	822.24
	80	1053.083	1142.4	1131.133	1116.66	1092.17
	100	1315.382	1439.067	1422	1378.3	1361.42
	120	1580.234	1686.067	1670.333	1681.68	1659.66
**10**	20	210.8533	252.5333	245.5333	231.12	225.17
	40	419.8867	485.5333	476.1333	447.48	425.86
	60	625.56	708.2667	688.6667	648.15	632.34
	80	835.12	925.8667	903.1333	874.4	862.33
	100	1041.54	1141.533	1126.467	1121.53	1077.6
	120	1249.073	1351.667	1336.333	1335.31	1314.26
**12**	20	174.5889	241.8667	234.2	230.24	181.23
	40	346.9334	448.1333	436.9333	433.83	380.85
	60	519.2055	597.3333	573.4667	567.99	537.33
	80	690.4666	790.0667	773.1333	765.2	725.21
	100	863.5278	988.6667	968.7333	960.43	927.43
	120	1034.789	1138.733	1113.4	1122.2	1102.28

**Table 6:** Experimental results for 15 replicated runs of SADP, GADP2, SOS, and SOS-LPT algorithms.

		SADP				GADP2				SOS				SOS-LPT			
Machines	Jobs	Min	Avg	Max	StDev	Min	Avg	Max	StDev	Min	Avg	Max	StDev	Min	Avg	Max	StDev
2	20	1196	1255	1338	33.80	1242	1254	1266	6.03	1152	1224	1260	30.48	1152	1201	1236	20.80
	40	2371	2462	2550	35.90	2441	2459	2474	8.26	2363	2378	2393	9.52	2360	2376	2393	10.60
	60	3588	3680	3764	41.20	3652	3675	3695	10.59	3519	3566	3631	32.86	3519	3557	3631	26.34
	80	4753	4879	5045	59.10	4846	4872	4896	11.89	4705	4735	4754	11.44	4705	4725	4754	13.85
6	20	441	455	481	8.80	448	454	459	3.08	380	386	393	3.77	381	386	393	3.78
	40	796	841	892	18.53	809	831	853	11.00	734	750	763	9.08	734	741	750	4.01
	60	1210	1259	1295	15.50	1219	1246	1270	12.05	1104	1118	1167	16.04	1096	1106	1113	5.56
	80	1606	1662	1705	18.80	1622	1648	1672	12.47	1500	1506	1514	4.91	1491	1503	1514	6.87

The PD plots shown in Fig 3, illustrate the relative deviation of the four algorithms from LB in all instances for each problem combination. The solutions obtained from the calculation of LB using Eq (11) above, were considered to be the optimum solution for which other algorithms were evaluated. However, the results of the relative deviation clearly show that the SOS-LPT implementation outperformed all the other methods. Next in the performance hierarchy is the SOS, followed by the ACOII approach.

**Fig 4:**
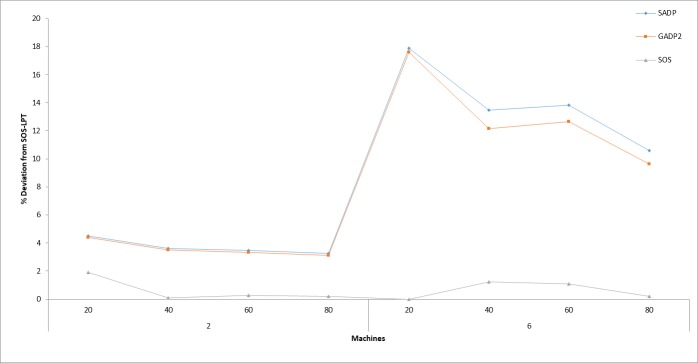
Percentage deviation of each algorithm with SOS-LPT as the control algorithm.

Fig 4 illustrates the results of the average percentage deviation of SADP, GADP2, and SOS algorithms from SOS-LPT, used here as the control algorithm, because of its excellent performance. Similarly, this figure provides an idea of which algorithm among the four methods performed better.

It is clearly seen that the initial claim that the proposed SOS algorithm is better than the other methods is justifiable if the computed average PD (%) values of each method are compared. Therefore, since the average PD (%) value obtained by the SOS-LPT which is 3.57%, being the least among the other two compared methods (10.89% for ACO and 9.26% for ACOII respectively), it can be concluded that the proposed methods are better alternatives than the other existing algorithms that address the same problem. It is also highlighted that the observed wide discrepancy between the high quality solutions obtained by the SOS-based methods (SOS and SOS-LPT) and other methods, suggests that the algorithm may be more appropriate for handling large problem instances.

## Statistical analyses

In order to conclude the whole analysis of the presented results and with the aim of making thorough analysis, there is need to further evaluate the statistical significance of our claims on the better performance of SOS and SOS-LPT. As such, two statistical tests are carried out. These tests include the paired-samples t-test and Friedman’s test with SOS-LPT used as the control algorithm.

### Paired samples t-test

To draw an unbiased conclusion for the above conducted experiments, a hypothesis test was performed to demonstrate the superiority of the proposed algorithms over the other existing methods. A paired-samples t-test was conducted to compare the average *C*_*max*_ values generated by SOS and SOS-LPT algorithms.

### Hypothesis

SOS-LPT versus SOS in terms of average *C*_*max*_ values

*H*_0_: *μ*_1_ = *μ*_2_, meaning that the average *C*_*max*_ values of SOS-LPT and SOS are the same.

*H*_1_: *μ*_1_ ≠ *μ*_2_ meaning that the average *C*_*max*_ values of SOS-LPT and SOS are not the same.

The paired samples correlation information shows that SOS and SOS-LPT average *C*_*max*_ values are significantly positively correlated (*r* = 1.000, *p* < 0.001). There was a significant average difference between SOS-LPT’s and SOS’s *C*_*max*_ (*t*_27_ = 7.465, *p* < 0.001). These results suggest that SOS average *C*_*max*_ values were 13 points higher than that of the SOS-LPT average *C*_*max*_ values (95% CI [9.47, 16.62]), where CI denotes confidence interval. Therefore, in this case, the null hypothesis *H*_0_ is rejected. Specifically, the test results suggest that SOS-LPT is statistically significantly better than the SOS in terms of average *C*_*max*_ values generated for all the problem instances considered.

### Friedman test

Friedman test was performed to further demonstrate the superiority of the SOS and SOS-LPT over other benchmarked algorithms. The ranking results obtained from the analyses of all the problem instance combinations are presented in Tables 7 and 8. The performance ranking results presented in Table 7 show the comparison between ACO, ACOII, SOS and SOS-LPT, while the results presented in Table 8, show the comparisons between GADP, SADP, SOS, and SOS-LPT. However, analysis and interpretation of the Friedman’s tests performed reveals that there is statistically significance difference among the evaluated algorithms with χ^2^ (3) = 90.300, *p* = 0.001, but the test does not exactly show where those differences lie. Therefore, Post hoc analysis with Wilcoxon signed-rank tests was further conducted with a Bonferroni correction applied, resulting in a significance level set at *p* < 0.0125. The Friedman test with post hoc tests results show that SOS-LPT is the overall best performed algorithm with (Z = -5.232, *p* = 0.001), and is closely followed by the SOS, while GADP appears to be the least performed method.

**Table 7:** Friedman’s rank test for the 180 instance combination of the benchmarked problem of ACO, ACOII, SOS, and SOS-LPT using their average *C*_*max*_ values.

Algorithm	Mean Rank	Rank
ACO	3.94	4
ACOII	2.69	3
SOS	2.28	2
SOS-LPT	1.08	1

**Table 8:** Friedman’s rank test for the 120 instance combination of the benchmarked problem of GADP, SADP, SOS, and SOS-LPT using their average *C*_*max*_ values.

Algorithm	Mean Rank	Rank
GADP	4.00	4
SADP	3.00	3
SOS	2.00	2
SOS-LPT	1.00	1

Finally, we can conclude by stating that under the same experimental settings and conditions, the two main contributions discussed in this paper, namely SOS and SOS-LPT, outperform the other alternative methods by showing better robustness and efficiency. In addition, the improvements shown by these two methods are significant in most cases and even more pronounced in the case of SOS-LPT that has been shown to be more superior to others. For this reason, we can say that the presented SOS and SOS-LPT are promising alternative methods to solve the UPMSP and its variants. While the conducted tests show very promising results for the proposed algorithms, it is noteworthy to mention here that the two algorithms (ACO and ACOII) were compared using balanced processing and setup times (which means that both the processing times and setup times were generated from the same uniform distribution) with data sets obtained from Scheduling Research Virtual Center (http://schedulingresearch.com/).

## Conclusion

This paper considers the implementation and application of an improved symbiotic organisms search optimization algorithm, to solve the parallel machine scheduling problem with the objective of minimizing makespan. The proposed approach resulting from the research, involves a two stage solution, which includes the use of the longest processing time first heuristic to generate an initial schedule of jobs to machine assignment for *n* jobs on *m* machines, and the employment of the improved SOS algorithm (SOS-LPT) to perform a global search update on the generated job sequence. In order to apply SOS-LPT to solve the unrelated parallel machine scheduling problem, a new encoding scheme was designed to increase the effectiveness of the SOS algorithm to handle the one-to-one mapping between candidate solution and individual organisms. The incorporation of the local search improvement mechanism into the SOS scheme has been shown to introduce diversity in the search process and avoid premature convergence. The performance of SOS-LPT is evaluated in comparison with previous results from other existing scheduling techniques from the existing literature. The experimental results clearly show that SOS-LPT substantially outperforms the other methods for all the problem instances tested. Future research suggested is to focus on developing an improved hybrid SOS for solving UPMSP that could handle both small and large scale instances efficiently. For example, it would be interesting and could be useful to extend this work by integrating 2-opt, 3-opt, or k-opt local search algorithms and investigate further for any possible improvement that can be accomplished using other approaches. The researchers plan to extend their research by incorporating additional improvements into the SOS by using hybridization techniques to include other meta-heuristics such as simulated annealing, and test its performance on different set of larger problem instances.
